# Crosstalk of Diabetic Conditions with Static Versus Dynamic Flow Environment—Impact on Aortic Valve Remodeling

**DOI:** 10.3390/ijms22136976

**Published:** 2021-06-28

**Authors:** Jessica I. Selig, Joana Boulgaropoulos, Naima Niazy, D. Margriet Ouwens, Karlheinz Preuß, Patrick Horn, Ralf Westenfeld, Artur Lichtenberg, Payam Akhyari, Mareike Barth

**Affiliations:** 1 Department of Cardiac Surgery, Medical Faculty, University Hospital Düsseldorf, Heinrich-Heine-University Düsseldorf, Moorenstraße 5, 40225 Düsseldorf, Germany; Jessica.Selig@med.uni-duesseldorf.de (J.I.S.); Joana.Boulgaropoulos@med.uni-duesseldorf.de (J.B.); Niazy.Naima@gmail.com (N.N.); Artur.Lichtenberg@med.uni-duesseldorf.de (A.L.); Mareike.Barth@med.uni-duesseldorf.de (M.B.); 2 Institute of Clinical Biochemistry and Pathobiochemistry, German Diabetes Center (DDZ), Auf’m Hennekamp 65, 40225 Düsseldorf, Germany; Margriet.Ouwens@ddz.de; 3 German Center for Diabetes Research (DZD), Ingolstädter Landstraße 1, Neuherberg, 85764 München, Germany; 4 Department of Endocrinology, Ghent University Hospital, Corneel Heymanslaan 10, 9000 Ghent, Belgium; 5 Faculty of Biotechnology, Bioprocessing, Modulation and Simulation, University of Applied Sciences Mannheim, Paul-Wittsack-Straße 10, 68163 Mannheim, Germany; K.Preuss@hs-mannheim.de; 6 Department of Cardiology, Pneumology and Angiology, Medical Faculty, University Hospital Düsseldorf, Heinrich-Heine-University Düsseldorf, Moorenstraße 5, 40225 Düsseldorf, Germany; Patrick.Horn@med.uni-duesseldorf.de (P.H.); Ralf.Westenfeld@med.uni-duesseldorf.de (R.W.)

**Keywords:** aortic valve stenosis, calcific aortic valve disease, fibrosis, diabetes mellitus, hyperinsulinemia, hyperglycemia, insulin signaling, 3D tissue culture, bioreactor, static tissue culture

## Abstract

Type 2 diabetes mellitus (T2D) is one of the prominent risk factors for the development and progression of calcific aortic valve disease. Nevertheless, little is known about molecular mechanisms of how T2D affects aortic valve (AV) remodeling. In this study, the influence of hyperinsulinemia and hyperglycemia on degenerative processes in valvular tissue is analyzed in intact AV exposed to an either static or dynamic 3D environment, respectively. The complex native dynamic environment of AV is simulated using a software-governed bioreactor system with controlled pulsatile flow. Dynamic cultivation resulted in significantly stronger fibrosis in AV tissue compared to static cultivation, while hyperinsulinemia and hyperglycemia had no impact on fibrosis. The expression of key differentiation markers and proteoglycans were altered by diabetic conditions in an environment-dependent manner. Furthermore, hyperinsulinemia and hyperglycemia affect insulin-signaling pathways. Western blot analysis showed increased phosphorylation level of protein kinase B (AKT) after acute insulin stimulation, which was lost in AV under hyperinsulinemia, indicating acquired insulin resistance of the AV tissue in response to elevated insulin levels. These data underline a complex interplay of diabetic conditions on one hand and biomechanical 3D environment on the other hand that possesses an impact on AV tissue remodeling.

## 1. Introduction

Calcific aortic valve disease (CAVD) is a progressive disorder that is characterized by a fibrotic thickening of the valve leaflets and remodeling of the extracellular matrix (ECM), as well as calcium accumulation in the valvular tissue [1]. These morphological alterations result in an immobilization of the leaflets that further results in regurgitation or stenosis of the aortic valve (AV). During this process, valvular interstitial cells (VIC), which is the main cell type of AV, are activated and display morphological and molecular changes [2]. Systemic factors, e.g., co-morbidities or pharmacological therapy, may have a modulating influence and contribute to the differentiation of the VIC. Several clinical studies indicate that commonly known cardiovascular risk factors and, in particular, the metabolic syndrome adversely affect the biological homeostasis of the AV [3,4,5,6,7,8]. Type 2 diabetes (T2D) has especially gained much attention as a disease-driving factor that is significantly associated with the increased risk of developing severe CAVD [6,7,8]. Moreover, T2D shows a steadily growing prevalence in industrialized countries and even more in middle-income and low-income countries, with considerable socio-economic consequences for health care systems [9,10,11].

Sustained high blood glucose levels known as hyperglycemia (HG) and alterations in insulin secretion, e.g., hyperinsulinemia (HI), characterize T2D. Insulin promotes intracellular activation of various phosphorylation cascades that affect metabolic and mitogenic processes, including protein synthesis, proliferation and apoptosis [12]. Impaired insulin sensitivity, as well as increased circulating insulin levels, favor a dysregulation in the latter processes due to an inadequate or overstimulated insulin response and may trigger pathological tissue changes resulting in secondary diseases [13,14,15].

In a previous in vitro study, we have shown that ovine VIC are sensitive to insulin. Interestingly, VIC exhibit increased proliferation under HI and also became insulin resistant due to diabetic cultivation conditions resulting in early degenerative changes [16]. Moreover, we observed that T2D is associated with increased expression of the proteoglycan biglycan in severely calcified human AV [17], substantiating the pro-degenerative role of biglycan in CAVD [18,19]. In order to increase our knowledge with respect to the methodological gap between in vitro studies involving VIC and studies on human valve explants, respectively, and also to shed light on the disease stages between those early changes observed in VIC and the final picture of calcified valves as observed in clinical specimens, further studies are required. In particular, the complexity of the dynamic three-dimensional (3D) environment of the native AV has been so far underexplored.

**Figure 1:**
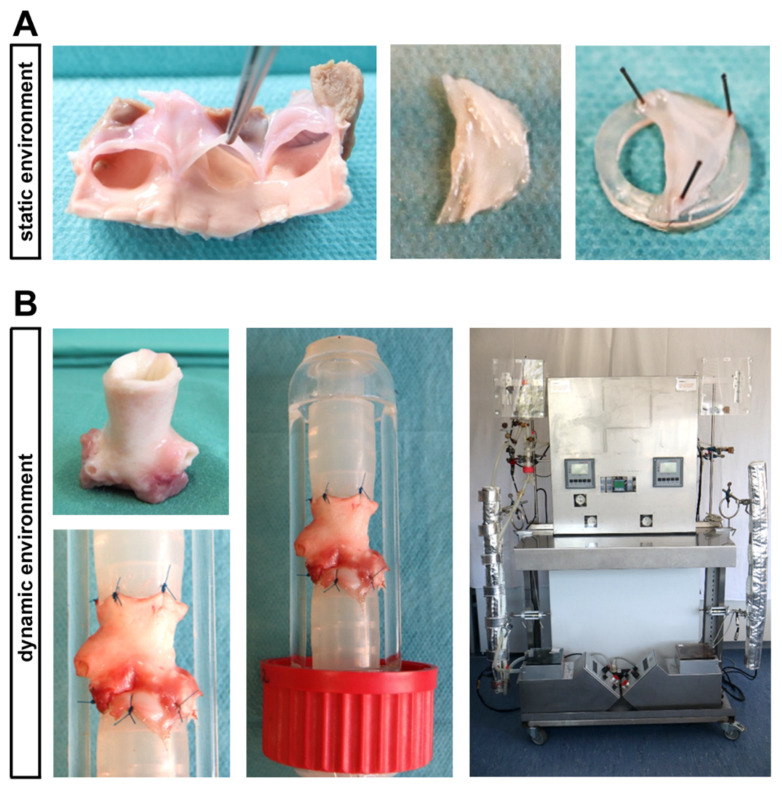
Ovine aortic valve (AV) cultivation in a static in vitro environment versus an ex vivo cultivation with dynamic flow in a bioreactor system. (**A**) In the static cultivation environment, AV leaflets were stretched with needles on a small silicone ring and were cultivated in culture plates. (**B**) The computer-assisted cultivation of whole AV conduits in the bioreactor system enables the simulation of physiological conditions ex vivo. In order to perform this, AV conduits were sewn between two silicone tubes, allowing a pulsatile flow of culture medium.

In front of this background, the current study has been designed to investigate the impact of T2D conditions on intact native AV tissue using a static in vitro culture environment as well as a controlled ex vivo setting comprised of dynamic flow conditions (Figure 1). Here, an elaborate bioreactor system allows a recapitulation of different components of the dynamic 3D environment of native AV, which are known to impact on homeostasis and degeneration of AV [20,21,22].

## 2. Results

### 2.1. Differentiation of VIC

**Figure 2:**
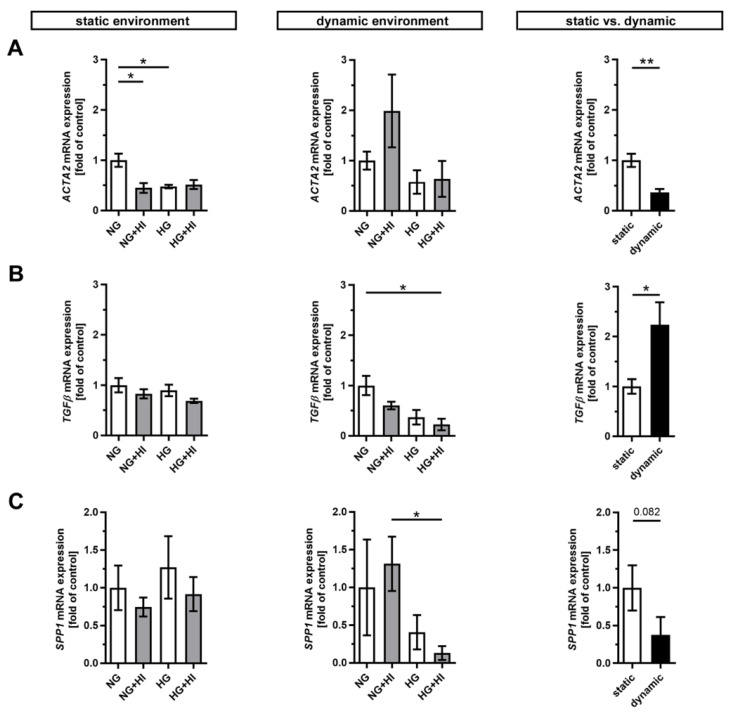
Gene expression of key differentiation markers in AV tissue. (**A**–**C**) Treatment with chronic HI or HG as well as the combination of those led in static cultivated AV leaflets to a decreased gene expression of α smooth muscle actin (*ACTA2*). Samples under dynamic condition of the bioreactor system in turn react with the downregulation of transforming growth factor β (*TGFβ*) and osteopontin (*SPP1*) under experimental diabetic conditions. Direct comparison of basal NG conditions showed a significant reduction in *ACTA2* and an increase in *TGFβ* gene expression under dynamic flow conditions compared to statically cultivated AV tissue, whereas *SPP1* gene expression remained unchanged. *n* = 5–6; *: *p* < 0.05; **: *p* < 0.01; NG: normoglycemia; HI: hyperinsulinemia; HG: hyperglycemia.

Pathological changes in AV tissue after ex vivo culture are associated with activation and differentiation of VIC and, thus, with the expression of key differentiation markers [2] (Figure 2). AV leaflets cultivated in a static environment displayed a significantly reduced expression of α smooth muscle actin (*ACTA2*) under NG + HI and HG cultivation conditions, respectively. This effect remained unaltered in samples cultured under dynamic flow conditions of the bioreactor system albeit with a strong variation under NG + HI (Figure 2A). In direct comparison of basal NG conditions, *ACTA2* expression was significantly decreased under dynamic flow conditions compared to the static environment (Figure 2A). Immunostaining, in contrast, showed a significantly higher integrated intensity under dynamic flow conditions (Supplemental Figure S1: ACTA2 immunostaining). On the other hand, the dynamic flow HG + HI conditions resulted in a decreased expression of transforming growth factor β (*TGFβ*) and osteopontin (*SPP1*). *TGFβ* expression was significantly higher and *SPP1* expression was reduced by a slight trend under dynamic cultivation when comparing basal NG conditions to static cultivation (Figure 2B,C).

### 2.2. Fibrosis of AV Leaflet Tissue

**Figure 3:**
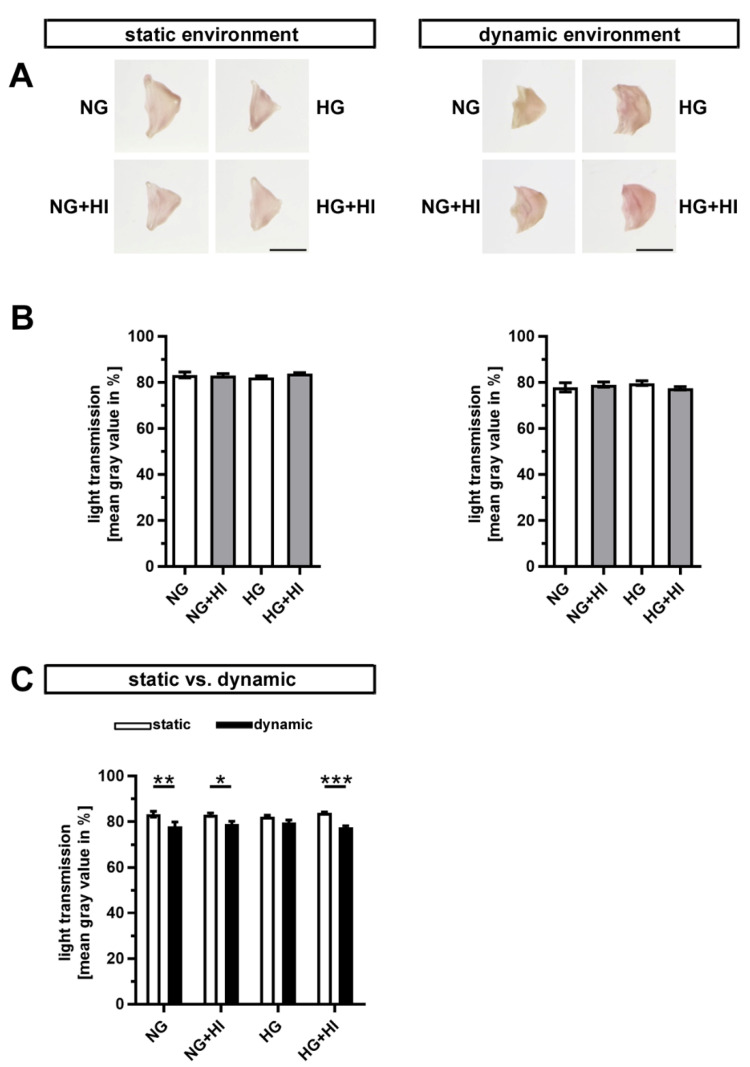
Density of AV tissue. (**A**) Macroscopic view of AV leaflets on a light pad after cultivation in a static (left) or dynamic flow environment (right). Bars: 1 cm. (**B**) Treatment of AV leaflets with chronic HI, HG or the combination of both had no impact on density of the tissue neither under static conditions (left) nor in an environment with dynamic flow (right). *n* = 5–6. (**C**) AV leaflets cultivated under dynamic flow conditions revealed a reduced light transmission and, with this, a higher density in comparison to statically cultivated AV tissue of the same treatment group. The calculated light transmission expressed as mean gray values inversely correlates with the level of tissue density in the sense that more dense tissue results in lesser light transmission and vice versa. *n* = 5–6; *: *p* < 0.05; **: *p* < 0.01; ***: *p* < 0.001; NG: normoglycemia; HI: hyperinsulinemia; HG: hyperglycemia.

Early stages of CAVD entail fibrosis of the AV, which results in a thickening and densification of the leaflets. In order to visualize tissue fibrosis, cultivated AV leaflets were photographed on a light pad and light transmission was calculated based on these images (Figure 3A). None of the conditions associated with T2D resulted in a significant change in fibrosis of AV leaflet tissue, irrespective of the applied static or dynamic condition, respectively (Figure 3B). However, AV samples cultured under dynamic flow conditions in direct comparison showed a significantly greater tissue density, which suggests a stronger activation of fibrotic processes under dynamic flow (Figure 3C).

**Figure 4:**
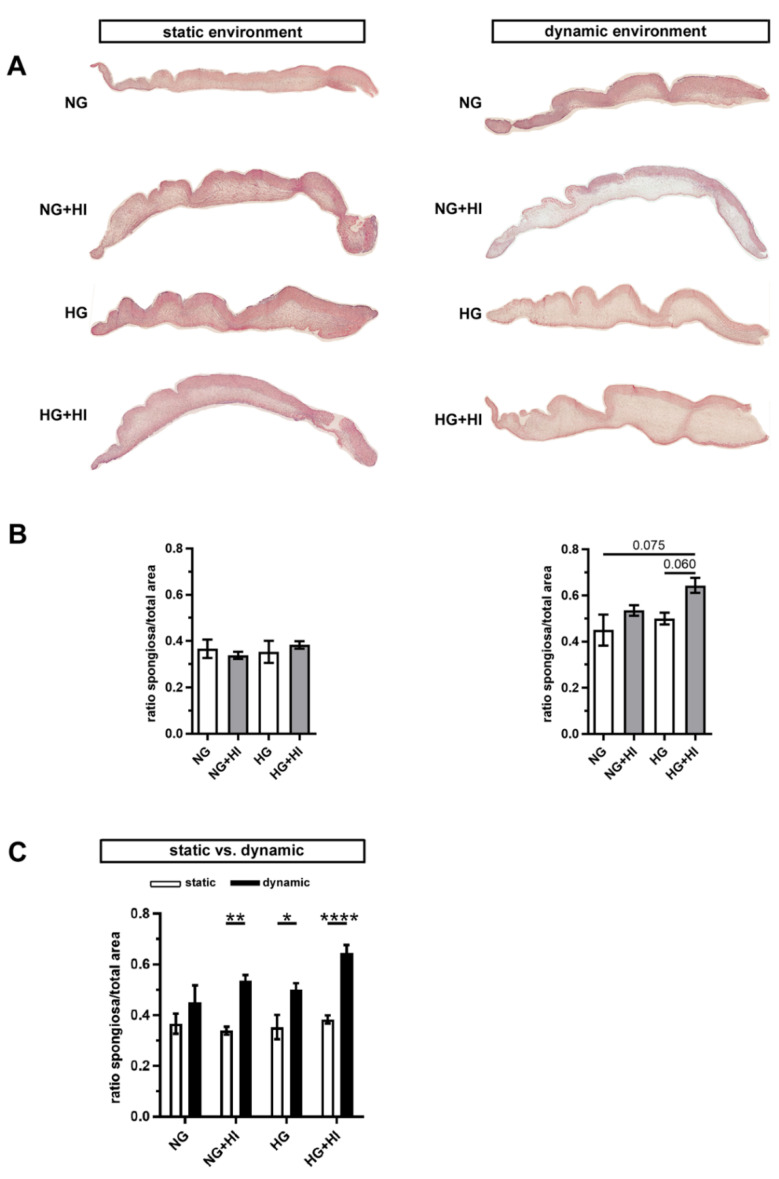
AV tissue remodeling. (**A**) Representative images of H&E staining of the cultivated leaflets with the aortic side up and the free edge to the left. Typical tissue architecture was preserved in all AV specimens comprising the three layers of AV: lamina fibrosa, lamina spongiosa and lamina ventricularis. (**B**) The ratio of lamina spongiosa area with respect to total leaflet area was calculated from leaflet cross sections as an indicator of tissue remodeling in the spongiosa in response to different cultivation conditions. While static samples were unaffected by chronic HI and HG, samples of the bioreactor system showed a trend towards the thickening of the lamina spongiosa due to HI + HG treatment. *n* = 5–6 (**C**) Dynamic flow cultivation resulted in increased hypertrophy in the spongiosa as compared to the static environment, irrespective of the applied metabolic condition. *n* = 5–6; *: *p* < 0.05; **: *p* < 0.01; ****: *p* < 0.0001; NG: normoglycemia; HI: hyperinsulinemia; HG: hyperglycemia.

In order to assess changes in the three-layered morphology of the AV as well as a thickening of the tissue, cross-sections were stained with hematoxylin and eosin (H&E) and further analyzed. In all AV, native tissue architecture comprised of three layers could be clearly identified. However, a macroscopic loosening of the matrix with a varying compactness was visible and found to particularly affect the lamina spongiosa (Figure 4A). In order to quantify this observation, the ratio between lamina spongiosa with respect to the total area of the leaflet was determined on the leaflet cross sections. While no differences were detectable in the static environment irrespective of the different settings of diabetic conditions (HG vs. HI vs. HG + HI), cultivation under dynamic flow conditions in the bioreactor system showed a trend towards thickening of the lamina spongiosa under HG + HI cultivation (Figure 4B). Marked differences were obvious in direct comparison of dynamic flow vs. static culture condition. When respective diabetic conditions were compared, bioreactor samples showed a significantly enlarged lamina spongiosa compared to the corresponding samples of static cultivation (Figure 4C).

### 2.3. ECM Remodeling

**Figure 5:**
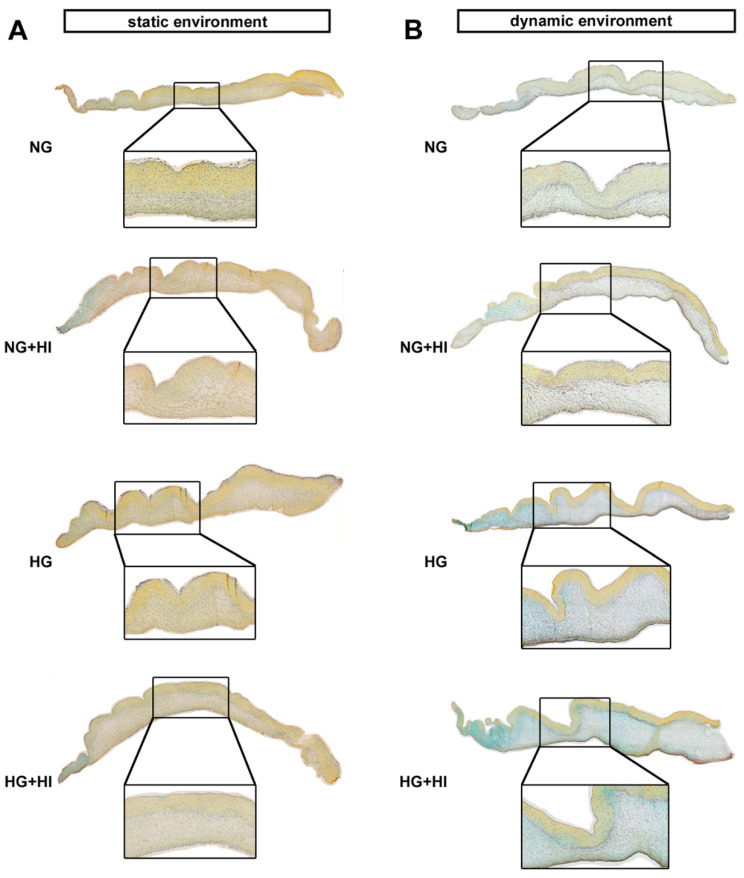
ECM composition of AV leaflets. (**A**,**B**) Representative images of Movat’s pentachrome staining of the cultivated leaflets with the aortic side up and the free edge to the left. Movat’s pentachrome stainings displayed a heterogeneous distribution of collagen (yellow) and proteoglycans (blue-green) in AV cross sections under the static (**A**) and dynamic environment (**B**). AV leaflets under dynamic conditions displayed stronger staining for proteoglycans (**B**). *n* = 5–6; NG: normoglycemia; HI: hyperinsulinemia; HG: hyperglycemia.

**Figure 6:**
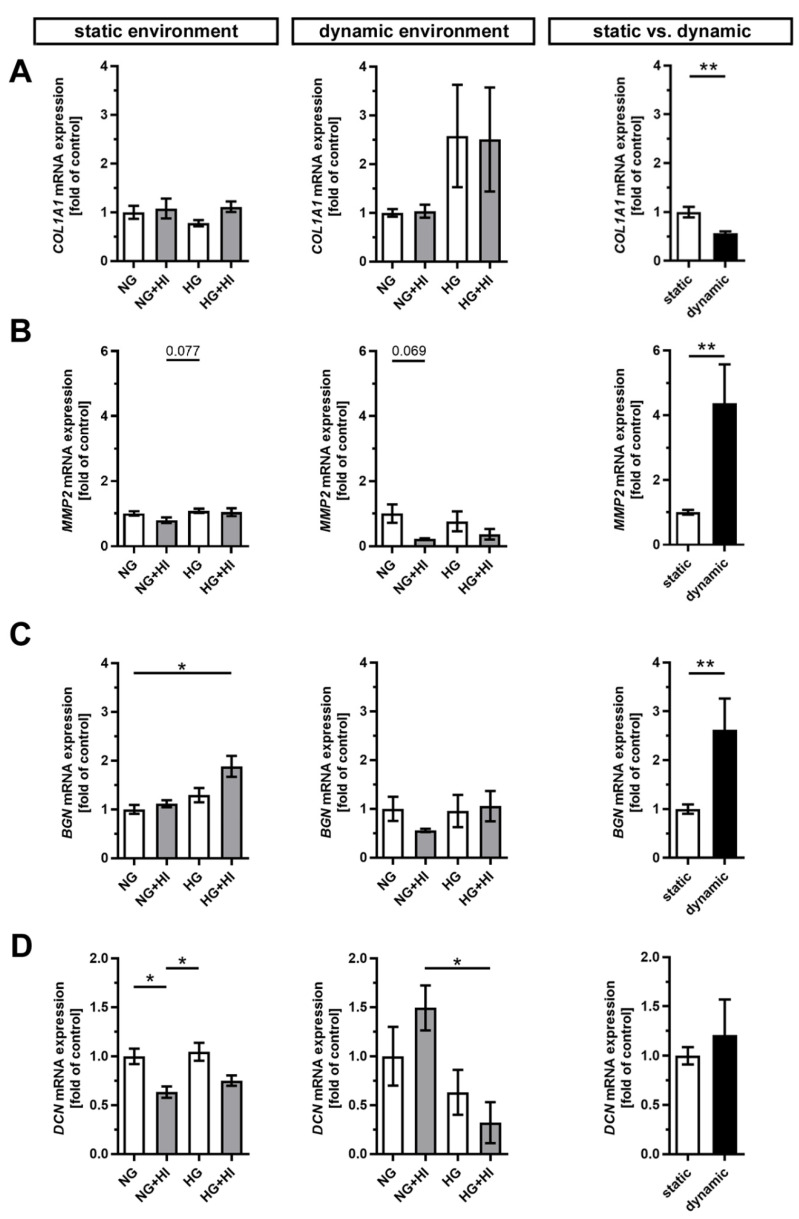
Gene expression of ECM molecules. (**A**–**D**) Gene expression of collagen type 1 alpha 1 (*COL1A1*) and its degrading enzyme matrix metalloproteinase 2 (*MMP2*) as well as the expressions of the small leucine-rich proteoglycans biglycan (*BGN*) and decorin (*DCN*) were influenced by chronic treatment with HI, HG and the combination of those in an environment-dependent manner. Direct comparison of basal NG conditions showed a significantly lower expression of *COL1A1* and significantly higher expressions of *MMP2* and *BGN* under dynamic flow conditions compared to statically cultivated AV tissue, whereas *DCN* expression remained unchanged. *n* = 5–6; *: *p* < 0.05; **: *p* < 0.01; NG: normoglycemia; HI: hyperinsulinemia; HG: hyperglycemia.

Development and progression of CAVD are accompanied by constant remodeling of the ECM, which is significantly modified by comorbidities, e.g., T2D [17,23]. Movat’s pentachrome staining of cultivated AV leaflets showed a local enrichment of collagen (yellow) and a heterogeneous distribution of proteoglycans (blue-green) across all herein analyzed groups, with a more prominent staining for proteoglycans under dynamic flow conditions (Figure 5). Under static culture, the expressions of collagen type I alpha 1 (*COL1A1*) and the collagen degrading enzyme matrix metalloproteinase 2 (*MMP2*) were more or less unchanged; a trend in decreased *MMP2* expression was observed only under NG + HI (Figure 6A,B). In samples of the dynamic flow environment, the expression of *COL1A1* seemed increased in both hyperglycemic groups (HG and HG + HI), while the expression of *MMP2* appeared to be lower in groups with chronic insulin treatment (NG + HI and HG + HI) although without statistical significance (Figure 6A,B). Direct comparison of basal NG conditions showed a significantly lower expression of *COL1A1* and a significantly increased expression in *MMP2* in the dynamic flow condition compared to the static condition (Figure 6A,B).

Furthermore, the expression of the proteoglycan biglycan (*BGN*) was significantly increased in statically cultivated leaflets under HG + HI conditions, while decorin (*DCN*) was downregulated in NG + HI and, by trend, reduced in HG + HI (Figure 6C,D). Under dynamic flow conditions, a seemingly diametrical regulation of the two proteoglycans *BGN* and *DCN* was observed. While *BGN* expression appeared to be diminished in leaflets under NG + HI, the expression of *DCN* was, by trend, elevated in this group (Figure 6C,D). Here, the direct comparison of basal NG conditions showed a significantly higher gene expression of *BGN* under dynamic flow conditions compared to static culture, whereas *DCN* remained unchanged (Figure 6C,D).

### 2.4. Activation of Insulin Pathways

**Figure 7:**
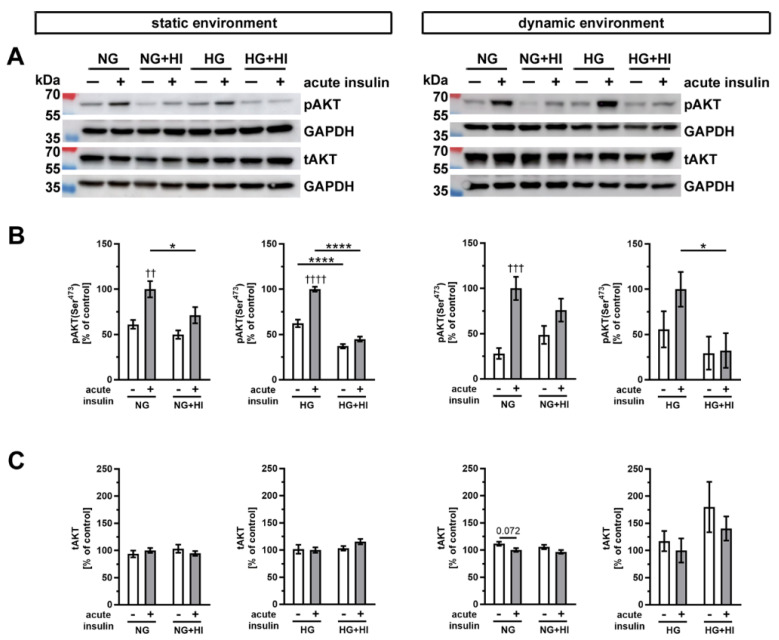
Activation of AKT signaling. (**A**) Representative Western blot images show the activation of AKT signaling due to diabetic cultivation conditions. Lanes of protein ladder represent 70 kDa and 55 kDa (pAKT and tAKT) as well as 35 kDa (GAPDH). (**B**) The effect of HI and HG on the insulin response of AV tissue became apparent by the phosphorylation level of AKT after acute insulin stimulation in comparison to the same condition without stimulation. The cultivation under different dynamic environments influenced the responsiveness of AV tissue to insulin. (**C**) The expression of total AKT was unaffected by the different diabetic conditions. Data were normalized to GAPDH and subsequently related to NG with acute insulin stimulus. *n* = 5–6; *: comparison between the indicated treatment groups; *: *p* < 0.05; ****: *p* < 0.0001; †: comparison according to the same condition without acute insulin stimulation; ††: *p* < 0.01; †††: *p* < 0.001; ††††: *p* < 0.0001; kDa: kilodalton; pAKT: phosphorylated protein kinase B; tAKT: total protein kinase B; GAPDH: glyceraldehyde 3-phosphate dehydrogenase; NG: normoglycemia; HI: hyperinsulinemia; HG: hyperglycemia.

Insulin-dependent signaling pathways were activated by an acute insulin stimulation (100 nM for 10 min), which results in phosphorylation of specific signal proteins involved in insulin action. One key component of insulin signaling is protein kinase B (AKT), which controls various intracellular downstream pathways by protein phosphorylation (Figure 7). Under normoglycemic controls (NG) of both cultivation environments, i.e., static as well as dynamic flow conditions, acute insulin stimulation induced a significant increase in phosphorylated AKT. This effect was lost in AV that were chronically exposed to elevated insulin levels during the cultivation period (NG + HI), which represents the development of insulin resistance in AV leaflets (Figure 7B). Under NG conditions in combination with either dynamic environment or static environment, acute insulin stimulation resulted in an increase in AKT phosphorylation levels. However, this relative change in AKT phosphorylation was smaller in the dynamic environment in comparison to the changes observed in the static environment. Under hyperglycemic conditions, comparable observations were made for AV in the static environment, i.e., pathological signaling response upon acute insulin stimulation. In contrast, samples cultured under dynamic flow conditions of the bioreactor system, in addition to HG, revealed no significant difference in response to acute insulin stimulation. Nevertheless, the general response patterns upon acute insulin stimulation were preserved in bioreactor groups under HG treatment. These findings may represent a hint for decreased insulin sensitivity of the tissue (Figure 7B). The total expression of AKT was, at the same time, not influenced by the acute insulin stimulation nor by chronic treatment with HI (Figure 7C).

**Figure 8:**
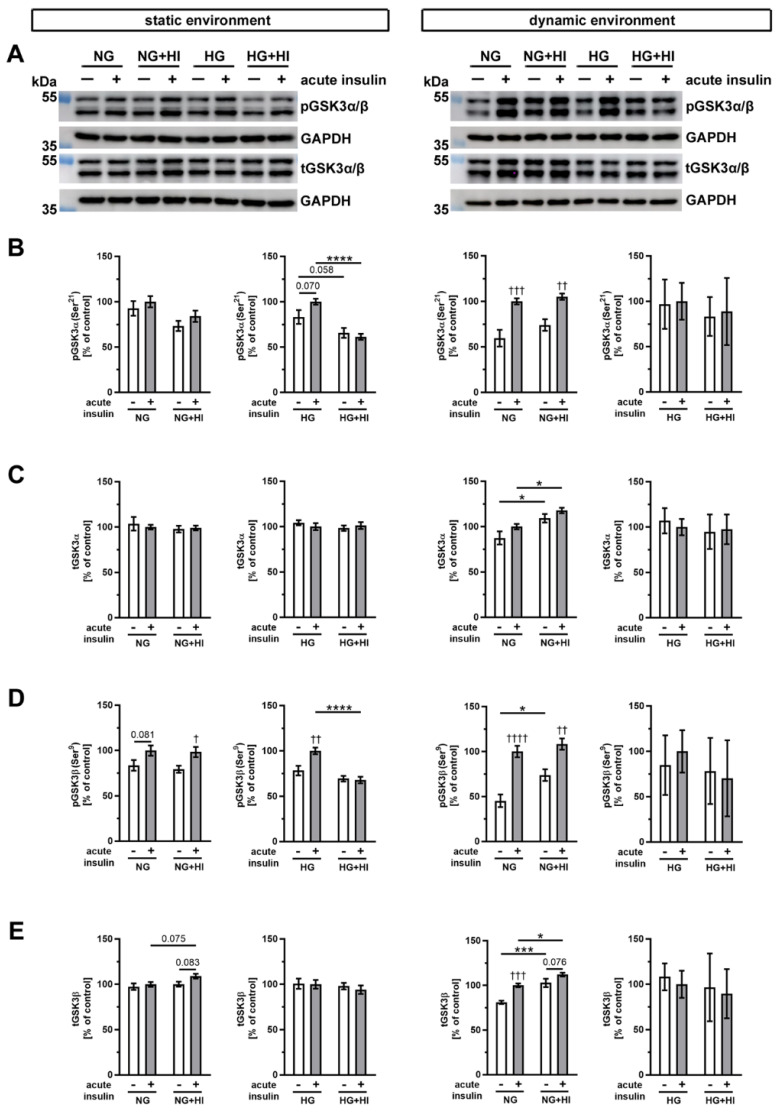
Activation of GSK3α/β signaling. (**A**) Representative Western blot images display levels of GSK3α/β phosphorylation under HI and HG and the respective expression of total GSK3α/β protein. Lanes of protein ladders represent 55 kDa (pGSK3α/β and tGSK3α/β) as well as 35 kDa (GAPDH). (**B**,**D**) Phosphorylation levels of GSK3α and GSK3β showed the insulin response of cultivated AV after acute insulin stimulation and were affected on the one hand by the chronic treatment with HI and HG and, on the other, by the cultivation environment (static versus dynamic flow). (**C**,**E**) Likewise, total GSK3α and GSK3β expressions of AV tissue were partially affected by diabetic conditions as well as the used cultivation environment. Data were normalized to GAPDH and subsequently related to NG with acute insulin stimulus. *n* = 3–6; *: comparison between indicated treatment groups; *: *p* < 0.05; ***: *p* < 0.001; ****: *p* < 0.0001; †: comparison according to the same condition without acute insulin stimulation; †: *p* < 0.05; ††: *p* < 0.01; †††: *p* < 0.001; ††††: *p* < 0.0001; kDa: kilodalton; pGSK3α/β: phosphorylated glycogen synthase kinase 3 α/β; t GSK3α/β: total glycogen synthase kinase 3 α/β; GAPDH: glyceraldehyde 3-phosphate dehydrogenase; NG: normoglycemia; HI: hyperinsulinemia; HG: hyperglycemia.

Glycogen synthase kinase 3 (GSK3), which exists in mammals in two isoforms GSK3α and GSK3β (Figure 8), is an important downstream target of AKT. In AV cultivated in a static environment, GSK3α was not affected by HI under NG conditions; however, under HG combined with HI conditions, a reduced phosphorylation level was observed (HG + HI; Figure 8B). At the same time, total GSK3α expression was stable (Figure 8C). In contrast, GSK3β showed much more robust phosphorylation response after acute insulin stimulation. While under NG this response was only by trend visible, we observed a significant increase in phosphorylation of GSK3β under NG + HI and under HG (Figure 8D). However, under diabetic conditions (HG + HI) the insulin-mediated activation of GSK3β was lost (Figure 8D).

After dynamic flow cultivation both under NG and under NG + HI cultivation conditions, we observed a significantly increased phosphorylation level of GSK3α and GSK3β after the acute insulin stimulation of AV leaflets (Figure 8B,D). The corresponding total GSK3α expression was independent of acute insulin stimulation, but revealed an elevated expression under NG + HI compared to NG alone (Figure 8C). Likewise, total GSK3β expression was significantly increased by chronic insulin treatment (NG + HI versus NG), with additional overexpression due to acute insulin stimulation (Figure 8E). In contrast, samples of the bioreactor system that were cultivated under hyperglycemic conditions (HG and HG + HI) showed no alterations in GSK3β abundance (Figure 8E).

**Figure 9:**
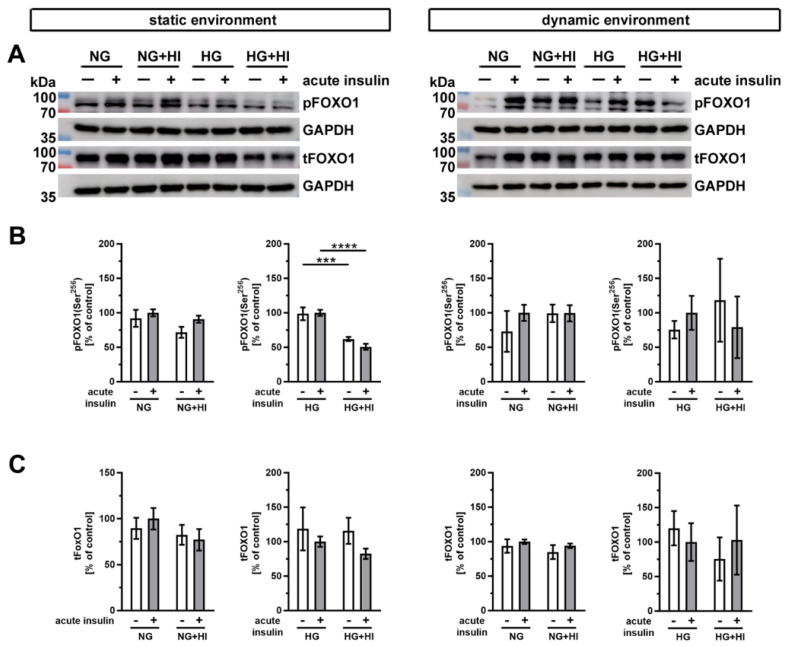
Activation of FOXO1 signaling. (**A**) Representative images of the Western blot analysis of FOXO1 show the phosphorylation level and respective total protein expression after chronic diabetic conditions under static environment versus dynamic flow cultivation. Lanes of protein ladders represent 70 kDa and 100 kDa (pFOXO1 and tFOXO1) as well as 35 kDa (GAPDH). (**B**) Significant effects of HI and HG treatment on the phosphorylation level of FOXO1 were only visible in statically cultivated AV tissue without and with acute insulin stimulation. (**C**) The expression of total FOXO1 was independent from chronic HI and HG treatment in both cultivation environments (static versus dynamic flow). Data were normalized to GAPDH and subsequently related to NG with acute insulin stimulus. *n* = 3–6; ***: *p* < 0.001; ****: *p* < 0.0001; kDa: kilodalton; pFOXO1: phosphorylated forkhead box protein O1; tFOXO1: total forkhead box protein O1; GAPDH: glyceraldehyde 3-phosphate dehydrogenase; NG: normoglycemia; HI: hyperinsulinemia; HG: hyperglycemia.

Another important substrate of AKT is the transcription factor forkhead box protein O1 (FOXO1) (Figure 9). Western blot analysis showed a significantly impaired phosphorylation level of FOXO1 in AV leaflets under HG + HI in comparison to HG, both without and with acute insulin stimulation (Figure 9B). Under normoglycemic conditions, we observed no differences in the FOXO1 phosphorylation, irrespective of static or dynamic environment (Figure 9B). Total FOXO1 abundance was not affected by diabetic cultivation conditions or by acute insulin stimulation (Figure 9C).

**Figure 10:**
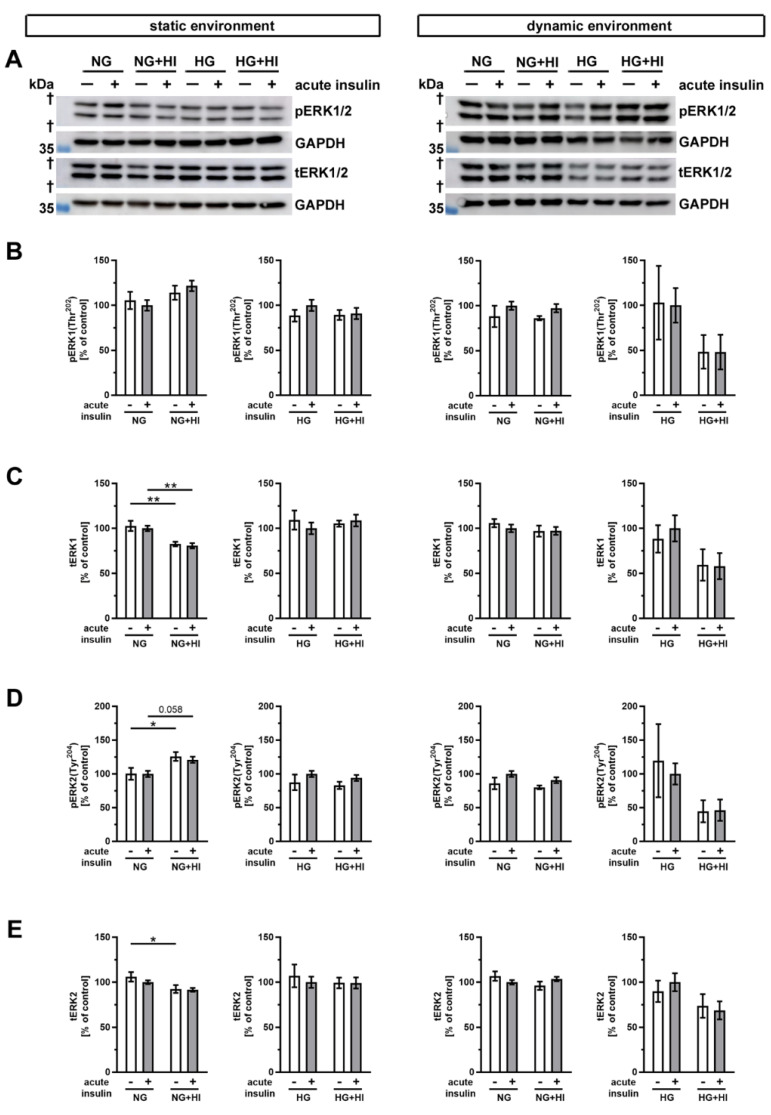
Activation of ERK1/2 signaling (**A**) Representative Western blot images demonstrate ERK1/2 phosphorylation as responses to acute insulin stimulation under diabetic cultivation conditions and its respective total ERK1/2 expression. Lanes of protein ladders represent 35 kDa (GAPDH). (**B**,**D**) While ERK1 phosphorylation was independent of chronic treatment with HI and HG in both utilized cultivation environments, ERK2 phosphorylation showed a regulation in statically cultivated AV leaflets. (**C**,**E**) Total protein expression of ERK1 and ERK2 was regulated in AV leaflets cultivated under static NG conditions, whereas HG and dynamic cultivation had no influence on the expression of total protein. Data were normalized to GAPDH and subsequently related to NG with acute insulin stimulus. *n* = 5–6; *: comparison between the indicated treatment groups; *: *p* < 0.05; **: *p* < 0.01; kDa: kilodalton; †: the presented bands localize between marker bands of 35 kDa and 55 kDa (out of display range); pERK1/2: phosphorylated extracellular signal-regulated kinase 1/2; tERK1/2: total extracellular signal-regulated kinase 1/2; GAPDH: glyceraldehyde 3-phosphate dehydrogenase; NG: normoglycemia; HI: hyperinsulinemia; HG: hyperglycemia.

Finally, we examined the phosphorylation of the mitogen-activated protein kinase 3/1 (ERK1/2) following insulin treatment (Figure 10). In static environments and under normoglycemic conditions, only slight alterations in ERK abundance were noted. Total abundance of ERK1 was decreased in AV tissue with HI in conditions without and with acute insulin stimulation (Figure 10B,C).

When ERK2 was considered, we observed a downregulation of the total abundance under HI, but phosphorylation level of ERK2 was increased. This suggests a selective activation of the signaling ERK2 pathway in the static condition (Figure 10D,E). However, the diabetic conditions had no impact on the total expression or phosphorylation level of ERK1 and ERK2 cultivation in the dynamic flow environment (Figure 10).

## 3. Discussion

The increasing prevalence and incidence of both T2D and CAVD represents a growing burden in Western developed countries, but the mechanisms by which T2D results in CAVD are incompletely understood. This work shows that the expression of early markers of CAVD is altered under diabetic conditions and that a specific 3D environment aggravates this detrimental change of expression profile. The results of this study demonstrate that in intact AVs, cusp tissue insulin-sensitive pathways are altered by diabetic conditions and may be further modified by the presence of dynamic flow conditions. This may result in a ‘vicious cycle’ in that insulin alters pathways towards structural alteration and fibrosis, resulting in an altered biomechanical environment which again fosters further detrimental remodeling. These findings suggest an interplay of metabolic dysregulation associated with T2D with local biomechanical factors to result in initiation and progression of CAVD.

### 3.1. Impact of Diabetic Conditions on Degeneration of Native AV Leaflets

In this work, intact AV leaflets in static cultivation seem to be quite resistant to degenerative changes induced by diabetic conditions. Neither macroscopic morphology (i.e., tissue density as a marker for tissue fibrosis) nor the expression of pro-degenerative markers were affected in our experiments. The expression of the differentiation marker *ACTA2* solely indicated a reduced myofibroblast phenotype of VIC as a response to cultivation under diabetic conditions. Under dynamic flow cultivation, *ACTA2* expression remained unchanged. However, unchanged *ACTA2* expression by HG is in line with our previous observations in VIC in a 2D culture model [16] and it is further in line with reports from other groups on VIC in a gelatin methacrylate 3D model [24] and also on cardiac fibroblasts [25]. Unaltered *ACTA2* expression in our bioreactor system might therefore be indicative for a balanced stress level. Increased basal gene expression of *ACTA2* in the static environment in direct comparison to the dynamic environment could be ascribed to enhanced stretch stress levels in this model, as it has been described before [26,27]. Differences in basal gene expression of *ACTA2* compared to basal protein abundance might be indicative for a diverse turnover dependent on biomechanical influence, as we have already observed for other targets in a similar setting [28]. 

Interestingly, *TGFβ* expression is unchanged under diabetic conditions in the static environment but reduced in the dynamic flow environment with the addition of diabetic conditions, especially under HG + HI treatment. A generally lower expression of *TGFβ* under static conditions might be the reason for the lack of susceptibility to diabetic conditions. Recent findings indicate that *TGFβ* expressions are generally higher in AV tissue cultivated under dynamic flow conditions than compared to a static cultivation environment, which is in line with our findings regarding direct comparison of basal conditions [28]. *TGFβ* is known to have an impact on VIC activation, i.e., *ACTA2* expression [29]. Thus, unchanged or even reduced *TGFβ* expression levels under static or dynamic flow conditions regarding diabetic treatment, respectively, might be the reason for reduced or unchanged *ACTA2* expression levels. Increased *TGFβ* expression levels in a dynamic environment under basal conditions reflect recent findings with a similar approach [28] and might contribute to the thickening of AV tissue by affecting extracellular matrix expression [30].

*SPP1* expression was unchanged under static conditions, which we have also observed in 2D cultivation under diabetic conditions [16]. In the dynamic flow environment, *SPP1* expression was reduced under HG and HG + HI treatment, but afflicted by high standard deviations. Others have reported the induction of *SPP1* and *TGFβ* in 3D models cultivating VIC under HG treatment. However, VIC in the latter model have been co-cultured with valvular endothelial cells [31]. Discrepancies between this model and our models thus might not only be due to mechanical differences but also might also be ascribed to the lack of VEC in our models, since VEC are known to communicate with VIC [32] possibly through protein kinase C under HG [24]. Direct comparisons of static vs. dynamic environment under basal conditions have shown a slight trend towards lower gene expression levels of *SPP1*, contrary to our previous observations in a similar setting [28]. Here, lower glucose levels in our basal conditions compared to the aforementioned approach using HG medium as basal medium might be the reason for this difference. Nevertheless, considering the elevated levels of *SPP1* gene expression in human fibrotic AV compared to unaltered AV [17], *SPP1* might not be the driving force for the thickening of AV leaflets in our biomechanical setting.

In general, dynamic flow cultivation resulted in fibrosis and thus results in the thickening of the tissue in comparison to samples under static cultivation independent of diabetic conditions. We mainly ascribe this to a generally higher *COL1A1* expression under dynamic flow cultivation compared to static cultivation, as we have observed this in a similar setting before [28]. Nevertheless, direct comparison of basal *COL1A1* expression levels in the present work show a significantly reduced expression under dynamic flow cultivation albeit within a small range, which might be attributed to lower glucose levels in our basal NG conditions compared to the aforementioned approach using HG medium as the basal medium [28]. Thus, *COL1A1* might not be exclusively responsible for the thickening of AV tissue under dynamic flow conditions. However, expression of ECM molecules also revealed cultivation environment-dependent differences in the reaction of AV to the respective diabetic condition. In both models, expression of *COL1A1* and *MMP2* remained largely unchanged, with only slight alterations by trend towards higher *COL1A1* expression and lower *MMP2* expression in bioreactor samples under diabetic conditions. Since 2D cultivation of VIC resulted in a significant increase in *COL1A1* expression under HG + HI treatment [16], this remodeling marker remains preserved in more complex 3D models. Nevertheless, other collagen types, such as collagen type 3 as well as MMP1/13 might be altered, since others have reported the induction of these markers by HG [24]. Independent from diabetic treatment, basal expression of *MMP2* was significantly higher under dynamic flow cultivation compared to static cultivation; this is indicative of an enhanced level of catabolic matrix degradation in AV tissue [33]. Further analysis of enzyme activity in the present setting would help to understand the impact of biomechanics since it has recently been shown that MMP2 activity correlates with shear stress [34]. 

Altered or increased proteoglycan expression due to diabetes or diabetic conditions has been reported in the context of cardiovascular disease (recently reviewed in [35]). In our previous work, we have shown that the proteoglycan *BGN* is upregulated in human degenerated AV of diabetics and in vitro in VIC under diabetic conditions in combination with pro-degenerative stimulus [17]. In the present work, static cultivation of AV under diabetic conditions (HG + HI) resulted in a similar effect, whereas AV under dynamic flow cultivation showed no alteration, suggesting a preservative influence of pulsatile flow on AV remodeling as induced by diabetic conditions. This effect might be due to the loss of proteoglycans under cyclic loading, as reported for cartilage tissue [36]. However, expression of *DCN* was mostly impaired by the influence of HI in the static cultivation approach and seems to be gradually reduced by diabetic conditions, in general, in the bioreactor approach. Nevertheless, basal levels of *BGN* but not of *DCN* were significantly higher under dynamic flow conditions, which might be responsible for a general enlargement of the lamina spongiosa under dynamic flow conditions. This observation is also in line with previous work showing increased expression levels of *BGN* in human fibrotic AV compared to unaltered AV, whereas *DCN* expression remained unchanged [17].

### 3.2. Insulin-Dependent Signaling in AV Leaflet Tissue

To our knowledge, the impact of diabetes or diabetic conditions on insulin signaling pathways in 2D or 3D models of CAVD has not been described in the literature so far. However, studies for classical insulin signaling have been reported for cardiac fibroblasts [25] and cardiomyocytes [37,38]. Binding of insulin to the insulin receptor triggers a phosphorylation cascade of downstream proteins, whereby the insulin signal is transmitted intracellularly [12]. In this process, protein kinase AKT represents a key component that controls various downstream pathways. In the present work, we could demonstrate that chronic insulin treatment resulted in reduced insulin signaling in the static 3D model. The latter negative impact of HI on insulin sensitivity was partly inhibited by the dynamic flow environment, especially under HG treatment. Previous studies with VIC in 2D culture presented similar results, even with stronger phosphorylation levels of AKT [16]. The altered amplitude in cell response may indicate a putative compensatory effect due to an interplay of VIC with ECM components when the intact AV leaflet tissue was examined. Downstream AKT signaling, however, revealed less pronounced insulin action in comparison to our experiences in the VIC 2D model. In the presented 3D models here, impaired insulin signaling visible by reduced phosphorylation of GSK3α/β under HI was mainly observed under HG treatment in static cultivation, but not inducible by dynamic flow cultivation. These heterogeneous responses to insulin might be affected by the general impact of mechanical stress on AKT/GSK3 signaling, as reported for cardiac fibroblasts [39] or pelvic fibroblasts [40]. Moreover, considering that our model represents a rather short-term approach, extended incubation might result in results that are more distinct. Experiments investigating the long-term effects of diabetic conditions will be necessary to ascertain this assumption.

In order to investigate potential signaling pathways and their impact on insulin signaling, we have also investigated FOXO1 phosphorylation. FOXO1 showed a rather weak reaction to diabetic conditions, whereas phosphorylation of FOXO1 under HG treatment under static cultivation showed a significant decrease by chronic insulin, indicating impaired insulin action putatively due to the strong diabetic stress when combining both HG and HI. From these findings, one may conclude that metabolic or mitogenic changes in AV leaflets may be largely independent of FOXO1. However, the transcriptional activity of FOXO1 is dependent on multiple phosphorylation residues regulated by a large number of kinases, which are also partly insulin sensitive [41,42]. More analyses are necessary for a better understanding of the involvement of FOXO1 in addition to the investigation of phosphorylation site Ser-256 that is conducted here.

Independent from the classical PI3K/AKT pathway, insulin signaling may be further transmitted via the RAS/MAPK pathway in cardiac diseases and diabetes [43,44]. Both downstream kinases, ERK1 and ERK2, showed only minor changes in AV leaflet tissue exposed to diabetic conditions with a trend towards impaired ERK2 phosphorylation by HI under NG conditions. Samples of the dynamic flow environment showed no regulation, whereas this might also be due to high standard deviations in the HG approach, requiring further investigations.

In the present models, we have examined two hallmarks of T2D as a first option to establish the models. However, our model does not reflect the clinical picture that is present in patients suffering from T2D. In order to approach a setting that more closely resembles T2D, the models could be augmented by components, such as pro-inflammatory cytokines or fatty acids, in future analyses. In addition, one may consider the analysis of expression patterns comparing the AV samples of the presented models with the AV of diabetics with an emphasis on the common characteristics and differences.

Considering the architecture of the native AV, future investigations could aim at the analysis of aspects of regional differences in biomechanics of the aortic valve leaflet itself. Certain regions of the native AV leaflet experience different ranges of shear stress and mechanical tension during cycles of opening and closure. Thus, the comparative analysis of gene or protein expression levels of AV leaflet regions originating, e.g., from the free edge, the leaflet center or the hinge region might elucidate the impact of the micromechanical environment on diabetes-induced AV degeneration.

### 3.3. Limitations of the Study

The standardized test conditions of tissue culture experiments enable a quick and reliable investigation of the fundamental characteristics of tissues and permits an effective analysis of external influencing factors. However, tissue cultures are artificial systems that only partially reflect the in vivo situation. AV tissue samples analyzed here are derived from hearts of six-month-old to eight-month-old lambs, which results in a certain discrepancy regarding the age of valve donors and the typical age of patients developing CAVD. Moreover, the clinical scenario of CAVD patients is frequently more complex as a number of comorbidities are present, which commonly affects the initiation and progression of CAVD [3,6,7]. Recent studies also indicate that the differentiation of non-human VIC in 2D cell cultures differs from that observed in patient samples, which suggests that our applied culturing parameters may not be sufficiently sophisticated to mimic the in vivo processes of CAVD in humans entirely [45,46]. Nevertheless, animal-derived tissues from local abattoirs are easy to obtain in large number and initially constitute a healthy phenotype that is probably less prone to biological variations compared to heart valve material potentially available from clinical sources, e.g., heart valve surgery.

Furthermore, tissue culture experiments are able to reflect the interplay between different cell types and the ECM, but they can only partially imitate the complex clinical pattern of many diseases. Here, the main characteristics of T2D, HI and HG were examined in a simplified manner and examined separately from systemic factors. In particular, tissue cultures do not take into account the involvement of activated immune cells and circulating cytokines that are associated with T2D and appear to be involved in initiating secondary diseases [47]. Nevertheless, the herein applied setup appears to be one of several promising strategies to investigate cause–effect principles for individual characteristics of a disease and to determine the specific influence of a given parameter, e.g., diabetic conditions. This may offer a basis for developing therapeutic approaches in the future. Collectively, ex vivo models of 3D tissue culture that consider mechanical stress in an integrative approach represent indispensable investigation models for translational research.

## 4. Materials and Methods

### 4.1. Ovine AV Tissue

Hearts from six to eight months old healthy sheep were obtained from a local abattoir (Laame GmbH & Co. KG, Wuppertal, Germany) and tissues were dissected within a maximum of four hours under sterile conditions.

### 4.2. In Vitro Cultivation under Static Conditions

For in vitro cultivation under static conditions, the three individual leaflets of an AV were prepared sterile from ovine hearts. In order to avoid the rolling in of the leaflets and to ensure a uniform rinsing with culture medium, the AV leaflets were stretched with three needles on 3 mm high silicone tubes (Figure 1A), as described previously [48,49]. The leaflets were cultivated in six-well-culture-plates with 10 mL DMEM culture medium (Thermo Fisher Scientific, Waltham, MA, USA), including 10% FCS (Sigma Aldrich, St. Louis, MO, USA) and 1% penicillin/streptomycin (Thermo Fisher Scientific) at 37 °C and 5% CO_2_. After pre-incubation for one day, cultivation under diabetic conditions was performed for seven days. Therefore, the tissue was cultivated either in DMEM culture medium with 100 mg/dl (normoglycaemia, NG) or 450 mg/dl glucose (hyperglycemia, HG) without or with 100 nM insulin (hyperinsulinemia, HI; Sigma Aldrich; cat. no. I5523). The culture medium was changed every second day in order to prevent a strong decrease in glucose concentration that has been shown to occur over time (Supplemental Figure S2: Necessity of medium changes; Supplemental Figure S3: Glucose concentration in culture medium).

### 4.3. Ex Vivo Cultivation in a Bioreactor System under Dynamic Flow Conditions

A computer-controlled bioreactor system allowed the cultivation of AV leaflets in their physiological valve anatomy together with pulsatile stretch (60 openings per minute) that is comparable to the heartbeat. Therefore, aortic roots including the AV were dissected under sterile conditions and pre-incubated in 25 mL DMEM culture medium including 10% FCS (Sigma Aldrich) and 1% penicillin/streptomycin (Thermo Fisher Scientific) at 37 °C and 5% CO_2_ for one day. Then, the AV conduits were sewn between two silicone tubes by stitches at the myocardium as well as on the aorta and inserted in the heart valve chamber of the bioreactor (Figure 1B). The cultivation was carried out in 550 mL culture medium including 10% FCS (Sigma Aldrich) and 1% penicillin/streptomycin (Thermo Fisher Scientific) under the same diabetic conditions as described for the static environment (NG, NG + HI, HG and HG + HI) for seven days. Several cultivation parameters, for instance, temperature, pH value, oxygen level or pressure on the AV were automatically documented and maintained constant by the bioreactor system. On the day of harvest, all three AV leaflets were dissected sterile out of the conduit, while adjacent myocardium and aorta were discarded.

### 4.4. Tissue Density

Tissue density of AV leaflets was measured by light transmission on a light pad (Slimlite LED 2447, Kaiser, Buchen, Germany). Therefore, all three leaflets of an AV were photographed in the dark under standardized conditions. Images were analyzed with ImageJ software version 1.50e (National Institutes of Health, Bethesda, MD, USA) by calculating the mean grey value of the tissue normalized to the mean grey value of the background. The lower the mean grey value was, the darker or rather denser the AV tissue was. Subsequently, the leaflets were used for further analyses.

### 4.5. Histochemical and Immunohistochemical Staining

In order to avoid a bias due to regional biomechanical differences of AV leaflets, one AV out of three AV leaflets of each replicate was bisected axially and perpendicularly to the free edge. Thus, one half was used for histochemical staining and the other half was conveyed to RNA analysis. AV tissue was cryopreserved in cryo-mounting medium (CryoCompound, Klinipath BV, Duiven, The Netherlands) and sections of 5 µm were prepared with a cryostat CM1950 (Leica Biosystems, Wetzlar, Germany). Histochemical staining was performed according to standard protocols for H&E and Movat’s pentachrome staining as described previously [50]. Images were acquired by a microscope system DM2000 (Leica Microsystems, Wetzlar, Germany) equipped with a digital camera DFC425 (Leica Microsystems) using Leica Application Suite software version 3.8 (Leica Microsystems) and by a Thunder Imager (Leica Microsystems) using the Leica Application Suite X (LAS X) software (Leica Microsystems). Semi-automatic morphometric analyses of the ratio between the area of lamina spongiosa and total area of the AV were performed with ImageJ software version 1.50e.

For immunohistochemical staining against ACTA2, tissue sections were fixed 10 min with ice-cold acetone. After washing steps in phosphate buffered saline (PBS), sections were incubated in 0.25% TritonX100 for 10 min and then blocked with 5% BSA/0.1% Tween in PBS for 60 min. Afterwards, the sections were incubated with the primary antibody (ab5694; Abcam, Cambridge, UK) for 60 min (1:300 in PBS) at room temperature. After washing in PBS, sections were incubated with 3% H_2_O_2_ for 10 min and washed again in PBS. Subsequently, sections were treated with a horseradish peroxidase-coupled secondary antibody (111-035-003; Dianova, Hamburg, Germany) for 45 min at room temperature. After washing steps in PBS, sections were treated with a DAB substrate kit (Zytomed, Berlin, Germany), according to the manufacturer’s instructions, and nuclei staining was performed using Meyer’s hematoxylin. Pictures were taken as described for histochemical staining and the quantification of integrated density was conducted using the Fiji/ImageJ2 software (see Supplemental Figure S1: ACTA2 immunostaining).

### 4.6. RNA Isolation and Determination of RNA Integrity

Total RNA was isolated with TRIzol reagent (Sigma Aldrich) according to the phenol chloroform method [51]. Therefore, snap-frozen tissue was disrupted mechanically with a tissue homogenizer (gentleMACS dissociator and M tubes, Miltenyi Biotec, Bergisch Gladbach, Germany). Afterwards, mRNA was purified with RNeasy Mini Kit (Qiagen, Venlo, The Netherlands) and treated with DNase (RNase-Free DNase Set, Qiagen), according to the respective manufacturers’ instructions. RNA integrity was determined by fluorescence-based capillary electrophoresis using Eukaryote Total RNA Nano Chips (Agilent Technologies, Santa Clara, CA, USA) in a Fragment Analyzer (Advanced Analytical Technologies, Heidelberg, Germany).

### 4.7. Gene Expression Analysis

For gene expression analysis, complementary DNA (cDNA) of the isolated mRNA was synthesized using the QuantiTect Reverse Transcription Kit (Qiagen). Gene expression was analyzed with semi-quantitative real-time PCR using GoTaq qPCR Master Mix (Promega, Madison, WI, USA) in a StepOnePlus Real-Time PCR System (Applied Biosystems, Waltham, MA, USA) according to standard protocols with StepOne Software version 2.3 (Applied Biosystems). Relative gene expression was determined by the comparative CT method (2^−ΔΔCT^ method; [52]) using 60S ribosomal protein L29 (*RPL29*), 60S ribosomal protein L13a (*RPL13a*) and β-tubulin (*TUBB*) as reference genes. The sequences of applied primer pairs are indicated in Table 1.

### 4.8. Protein Isolation and Quantification

Before harvesting, the AV leaflets were starved for 4 h in cultivation medium without FCS. Afterwards the leaflets of each replicate were bisected axially and perpendicularly to the free edge, whereas one half was stimulated with 100 nM insulin (acute insulin, AI) in PBS (Thermo Fisher Scientific) for 10 min to activate insulin-signaling pathways, while the other one remained in PBS without insulin as untreated condition. Appropriate conditions were evaluated in preliminary experiments (Supplemental Figure S4: Duration of acute insulin stimulation). After stimulation AV were snap-frozen, mechanically homogenized and solved in RIPA lysis buffer including PhosStop (Sigma Aldrich) and protease inhibitors (cOmplete, Sigma Aldrich). Cell disruption was carried out by sonification (Sonorex RK 156, Bandelin electronic, Berlin, Germany) for 15 min followed by centrifugation in order to remove the cell debris. The total protein concentration was determined by DC assay (Bio-Rad Laboratories, Hercules, CA, USA) according to the manufacturer’s instructions.

### 4.9. Western Blot Analysis

Proteins were separated together with the protein ladder (PageRuler Plus Prestained protein Ladder, Thermo Fisher Scientific) on 10% reducing sodium dodecyl sulfate-polyacrylamide gels and blotted on nitrocellulose membranes (Bio-Rad Laboratories). For protein detection, the following primary antibodies were used: phospho-AKT(Ser473) (Cell Signaling Technology, Danvers, MA, USA; cat. no. 4060); total AKT (Cell Signaling Technology; cat. no. 4691); phospho-GSK3α(Ser21)/β(Ser9) (Lifespan Biosciences, Seattle, WA, USA; cat. no. LS-C7154); total GSK3α/β (Cell Signaling Technology; cat. no. 5676); phospho-FOXO1(Ser256) (Cell Signaling Technology; cat. no. 9461); total FOXO1 (Cell Signaling Technology; cat. no. 2880); phospho-ERK1(Thr202)/2(Tyr204) (Cell Signaling Technology; cat. no. 4370); and total ERK1/2 (Santa Cruz Biotechnology, Dallas, TX, USA; cat. no. sc-514302). Signal detection was performed with horseradish peroxidase-coupled antibodies against mouse (Jackson ImmunoResearch Laboratories, West Grove, PA, USA; cat. no. 115-035-044) or rabbit (Jackson ImmunoResearch Laboratories; cat. no. 111-035-003) and WesternBright Chemilumineszenz Substrat Quantum (Biozym, Hessisch Oldendorf, Germany) imaged with an Amersham Imager 600 (GE Healthcare, Chicago, IL, USA). Signal intensities were measured with ImageQuant TL software version v8.1.0.0 (GE Healthcare) and adjusted by the background signal of the membrane. Target protein signals were normalized to a glyceraldehyde 3-phosphate dehydrogenase (GAPDH) signal (Cell Signaling Technology; cat. no. 2118) detected on the same nitrocellulose membrane.

### 4.10. Statistical Analysis

Statistical analysis and graphical presentation were performed with GraphPad Prism version 6.01 (GraphPad Software Inc., La Jolla, CA, USA). Reported data are presented as mean ± standard error of mean. The Mann–Whitney U test was used for comparisons between two groups and for comparisons between more than two groups the Kruskal–Wallis test with Dunn’s multiple comparisons post hoc test was performed. Analyses of two independent variables were performed with two-way ANOVA and subsequent Sidak’s multiple comparisons post hoc test. *p*-values < 0.05 were considered as statistically significant different (*: *p* < 0.05; **: *p* < 0.01; ***: *p* < 0.001; ****: *p* < 0.0001).

## 5. Conclusions

Chronic hyperglycemia and hyperinsulinemia result in impaired insulin signaling in intact aortic valve tissue and includes features of insulin resistance. The presence of a dynamic environment has a modulating effect on the adverse changes induced by diabetic conditions. These observations suggest a relevant interplay of metabolic and biomechanical factors with divergent contributions to the initiation and progression of calcified aortic valve disease.

## Figures and Tables

**Table 1:** Primer sequences used for gene expression analysis.

Gene	Forward Sequence (5′ → 3′)	Reverse Sequence (5′ → 3′)
*ACTA2*	GATAGAGCACGGCATCATCA	GAAGGGTTGGATGCTCTTCA
*BGN*	TCTGCTCCGCTACTCCAAGT	TTGTTGTCCAAGTGCAGCTC
*COL1A1*	AAGACATCCCACCAGTCACC	TAAGTTCGTCGCAGATCACG
*DCN*	CCAAAGTGCGAAAGTCTGTG	TTCAATGCCTGAGCTCTTCA
*MMP2*	TGACAAGGACGGCAAGTATG	GTAAGATGTGCCCTGGAAGC
*RPL13a*	GATCCCACCACCCTATGACA	CTTCAGACGCACAACCTTGA
*RPL29*	CCAAGTCCAAGAACCACACC	TATCGTTGTGATCGGGGTTT
*SPP1*	GATGGCCGAGGTGATAGTGT	TCGTCTTCTTAGGTGCGTCA
*TGF* *β*	GAGCCAGAGGCGGACTACTA	TCGGACGTGTTGAAGAACAT
*TUBB*	CCTACAACTGGACCGCATCT	AAAGGACCTGAGCGAACAGA

## Data Availability

The data presented in this study are available upon reasonable request from the corresponding author.

## Supplementary Materials

The following are available online at https://www.mdpi.com/article/10.3390/ijms22136976/s1.
