# Supplementary material for: Crosstalk of Diabetic Conditions with Static Versus Dynamic Flow Environment—Impact on Aortic Valve Remodeling
Source: Int J Mol Sci. 2021 Jun 28;22(13):6976. doi: 10.3390/ijms22136976 (PMC8268732; doi:10.3390/ijms22136976)
Supplement: Supplementary file 1 [file ijms-22-06976-s001.zip › ijms-1236306-supplementary.pdf]

Supplemental figure S1

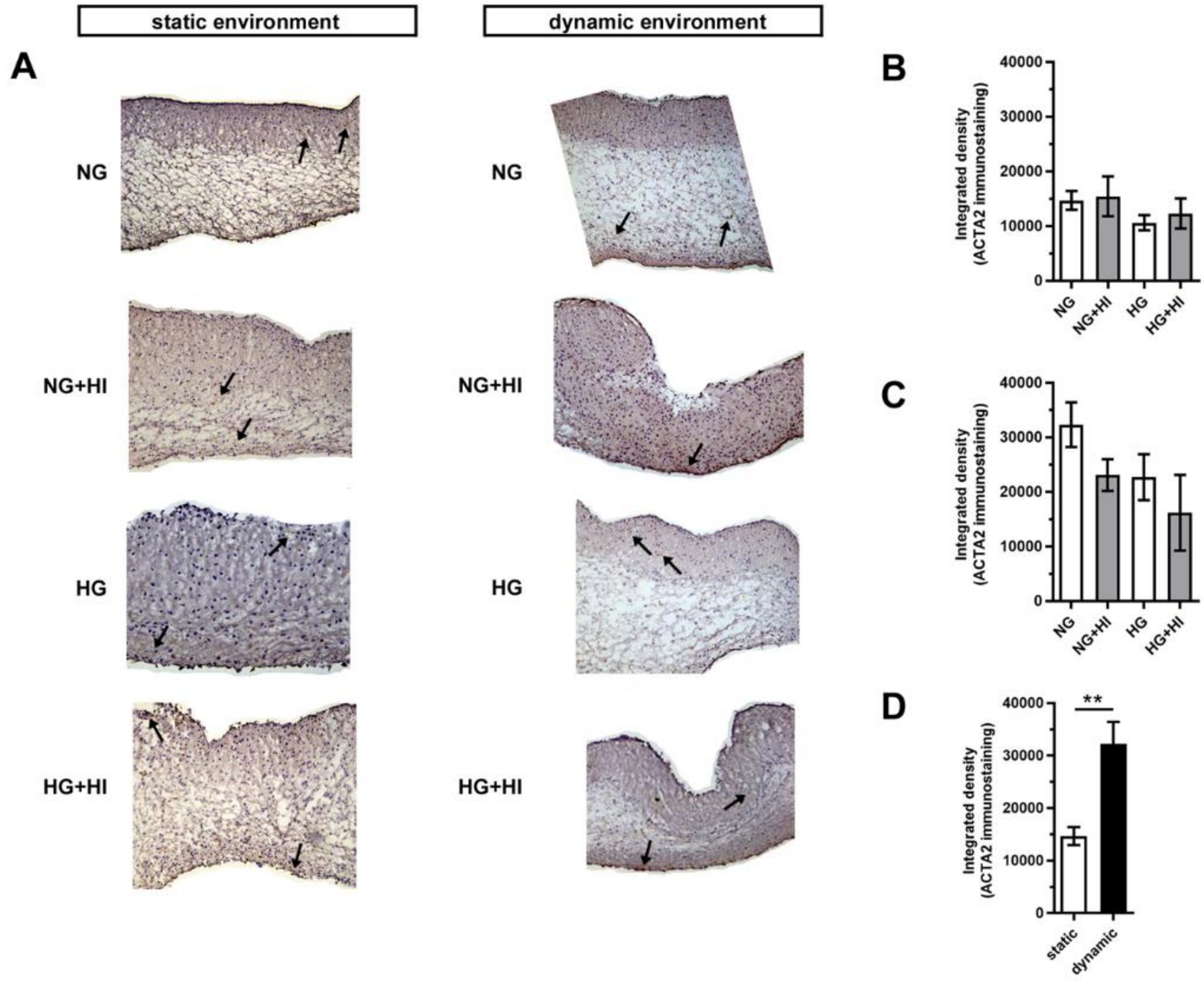

Figure S1. ACTA2 immunostaining. (**A**) Immunohistochemical staining against ACTA2 revealed no macroscopic differences in staining intensity due to diabetic treatment, neither under static nor under dynamic conditions. Cells staining for ACTA2 are loosely dispersed (see arrows). Quantification of staining intensity also showed no differences between the diabetic treatments, neither under static environment (**B**) nor under dynamic environment (**C**). Comparison of basal NG conditions showed a significant higher intensity of ACTA2 immunostaining under dynamic conditions compared to static conditions (**D**). n = 5; **: $p < 0.01$; NG: normoglycemia; HI: hyperinsulinemia; HG: hyperglycemia

Supplemental figure S2

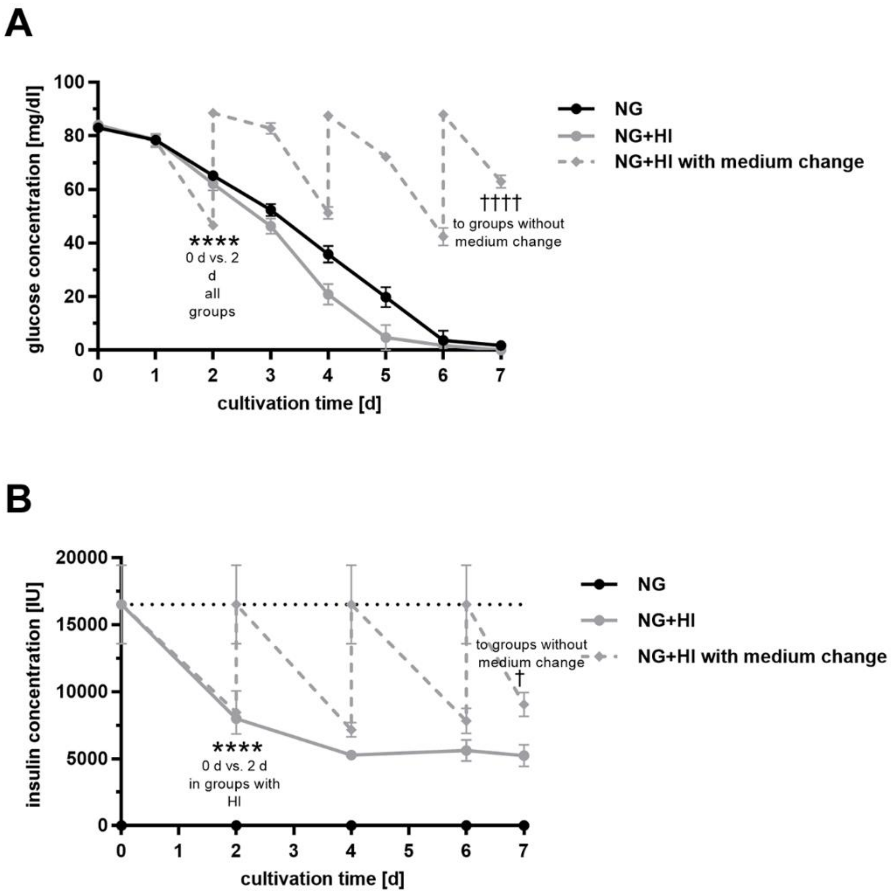

Figure S2. Preliminary experiments on the necessity of medium changes in the static environment. (**A**) Glucose concentration in the medium was measured with a blood glucose meter in duplicates. After two days, the glucose concertation decreased significantly in all groups and was in the groups without medium changes at day six under 10 mg/dl. Medium changes all two days ensured a more stable glucose concentration between 40 mg/dl and 100 mg/dl in the culture medium over the complete period of cultivation. n = 6. (**B**) Insulin concentration was measured all two days before medium changes with an insulin ELISA (Rat Insulin ELISA, RayBiotech, Peachtree Corners, GA, USA) and showed in both groups with HI a significant decrease after two days. In contrast to the glucose concentration, the insulin amount remained despite variations on a high level over the whole cultivation period. Nevertheless, NG+HI with medium change was significant higher compared to NG+HI without medium change on day of harvest. In the control group without HI, insulin was not detectable indicating that insulin included in the FCS is negligible. n = 6; ****: $p < 0.0001$ 0 d versus 2 d of the same treatment group; †: $p < 0.05$ to groups without medium change; ††††: $p < 0.0001$ to groups without medium change; NG: normoglycemia; HI: hyperinsulinemia; HG: hyperglycemia

Supplemental figure S3

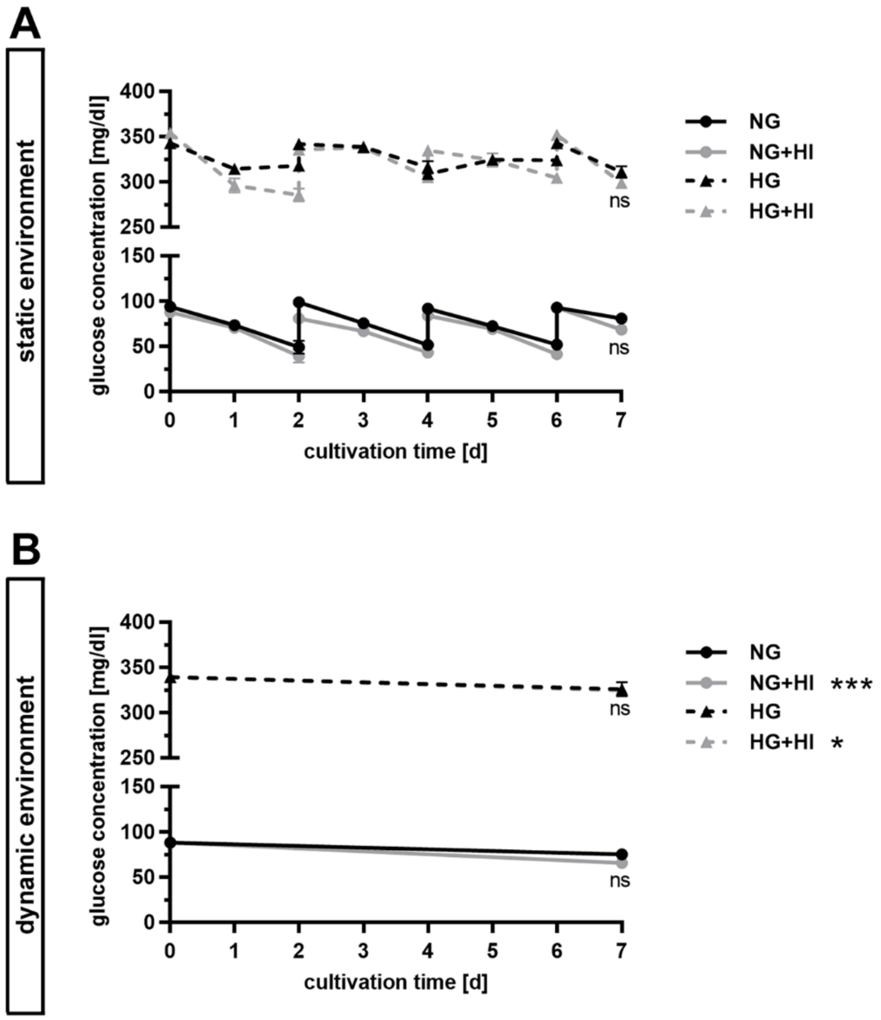

Figure S3. Glucose concentration in culture medium. (**A**) In the static environment, the glucose concentration fluctuates over the cultivation period. However, the glucose amount at day seven was not influenced by the treatment with HI in comparison to the according glucose control group. n = 6. (**B**) Also under dynamic flow cultivation HI treatment did not affect the glucose concentration on day of harvest, but led to a decrease in the glucose amount in comparison to the starting time point under NG as well as under HG conditions. n = 5; *: $p < 0.05$ 0 d versus 7 d of the same treatment group; ***: $p < 0.001$ 0 d versus 7 d of the same treatment group; NG: normoglycemia; HI: hyperinsulinemia; HG: hyperglycemia

Supplemental figure S4

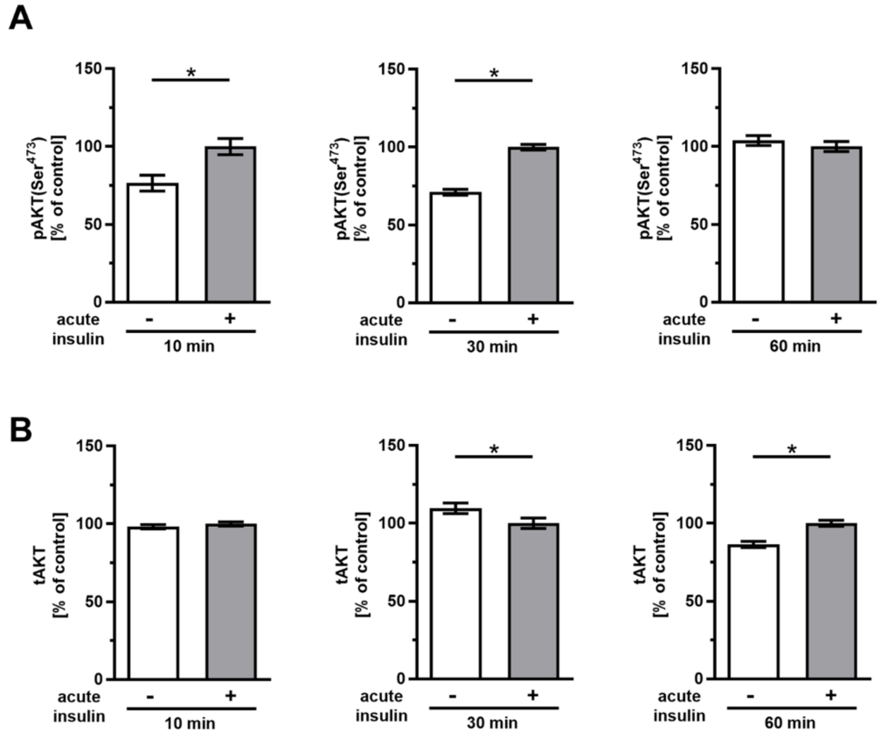

Figure S4. Preliminary experiments on the duration of acute insulin stimulation. **(A/B):** In order to ensure a sufficient activation of insulin-dependent signaling pathways, three different durations for acute insulin stimulation (10 minutes, 30 minutes and 60 minutes) were tested. Therefore, freshly prepared AV leaflets were pre-incubated for one day under static NG conditions, starved for 4 h in FCS-free culture medium and stimulated with 100 nM insulin for the corresponding period of time. Subsequently, the activation of insulin signaling was evaluated by the phosphorylation intensity of AKT. It was found that an acute insulin stimulation for 10 minutes is sufficient for a significantly increased AKT phosphorylation, while a longer stimulation leads to expression alterations in the total amount of AKT and/or a loss of insulin response. n = 4; *: $p < 0.05$; pAKT: phosphorylated protein kinase B; AKT: protein kinase B.
